# Comparative Evaluation of ARB Monotherapy and SGLT2/ACE Inhibitor Combination Therapy in the Renal Function of Diabetes Mellitus Patients: A Retrospective, Longitudinal Cohort Study

**DOI:** 10.3390/ijms26157412

**Published:** 2025-08-01

**Authors:** Andrew W. Ngai, Aqsa Baig, Muhammad Zia, Karen Arca-Contreras, Nadeem Ul Haque, Veronica Livetsky, Marcelina Rokicki, Shiryn D. Sukhram

**Affiliations:** 1 Department of Biology, College of Staten Island, City University of New York, 2800 Victory Boulevard, Building 6S, Staten Island, NY 10314, USA; andrew.ngai@cix.csi.cuny.edu (A.W.N.); veronica.livetsky@cix.csi.cuny.edu (V.L.); marcelina.rokicki@cix.csi.cuny.edu (M.R.); shiryn.sukhram@csi.cuny.edu (S.D.S.); 2 Department of Environmental and Public Health Sciences, University of Cincinnati, 160 Panzeca Way, Kettering Lab Building, Cincinnati, OH 45267, USA; baigaq@mail.uc.edu; 3 Nursing Department, College of Staten Island, City University of New York, 2800 Victory Boulevard, Building 5S, Staten Island, NY 10314, USA; karen.arcacontreras@csi.cuny.edu; 4 AZZ Medical Associates, 631 Broadway, Suite 2, Bayonne, NJ 07002, USA; haqueoffice@gmail.com

**Keywords:** diabetic nephropathy, diabetic kidney disease, angiotensin II receptor blockers, sodium-glucose cotransporter-2 inhibitors, angiotensin-converting enzyme inhibitors, chronic kidney disease

## Abstract

Diabetic nephropathy affects approximately 30–40% of individuals with diabetes mellitus (DM) and is a major contributor to end-stage renal disease (ESRD). While angiotensin II receptor blockers (ARBs) have long served as a standard treatment, sodium-glucose cotransporter-2 inhibitors (SGLT2i) have recently gained attention for their renal and cardiovascular benefits. However, comparative real-world data on their long-term renal effectiveness remain limited. We conducted a retrospective, longitudinal study over a 2-year period to compare the impact of ARB monotherapy versus SGLT2i and angiotensin-converting enzyme inhibitor (ACEi) combination therapy on the progression of chronic kidney disease (CKD) in patients with DM. A total of 126 patients were included and grouped based on treatment regimen. Renal biomarkers were analyzed using *t*-tests and ANOVA (*p* < 0.01). Albuminuria was qualitatively classified via urinalysis as negative, level 1 (+1), level 2 (+2), or level 3 (+3). The ARB group demonstrated higher estimated glomerular filtration rate (eGFR) and lower serum creatinine (sCr) levels than the combination therapy group, with glycated hemoglobin (HbA1c), potassium (K^+^), and blood pressure remaining within normal limits in both cohorts. Albuminuria remained stable over time, with 60.8% of ARB users and 73.1% of combination therapy users exhibiting persistently or on-average negative results. Despite the expected additive benefits of SGLT2i/ACEi therapy, ARB monotherapy was associated with slightly more favorable renal function markers and a lower incidence of severe albuminuria. These findings suggest a need for further controlled studies to clarify the comparative long-term renal effects of these treatment regimens.

## 1. Introduction

Diabetic nephropathy (DN), also referred to as diabetic kidney disease, is a leading cause of chronic kidney disease (CKD) worldwide and is clinically characterized by persistent albuminuria (typically defined as an albumin-to-creatinine [ACR] ratio exceeding 30 mg/g) and a progressive decline in kidney function. CKD is classified into five stages, culminating in end-stage renal disease (ESRD), a condition requiring dialysis or transplantation. It is estimated that 30 to 40 percent of individuals with diabetes mellitus (DM) develop CKD, highlighting the significance of DN as a global public health concern [1,2]. DN contributes substantially to morbidity and mortality, complicating clinical management and substantially reducing health-related quality of life [1].

Current therapeutic strategies aim to slow DN progression by modulating pathways involved in renal injury, most notably through inhibition of the renin–angiotensin–aldosterone system (RAAS). Agents such as angiotensin-converting enzyme inhibitors (ACEi) and angiotensin II receptor blockers (ARBs) have demonstrated efficacy in reducing serum creatinine (sCr) levels and urinary albumin excretion (UAE), thereby preserving renal function [3,4,5,6]. While both drug classes target the RAAS, comparative studies have revealed nuanced differences. Some data suggest that ACEi, particularly at higher doses, may offer superior reduction in UAE, whereas ARBs are generally better tolerated and are associated with fewer adverse effects such as cough or angioedema, which are attributed to bradykinin accumulation [7,8,9].

Meta-analyses of randomized trials further support the renoprotective effects of RAAS inhibition, especially in patients with type 2 diabetes (T2DM) and microalbuminuria (MAU) or overt proteinuria [3]. Furthermore, RAAS blockade has been shown to outperform other antihypertensive classes (e.g., beta-blockers, calcium channel blockers, and diuretics) in slowing CKD progression in diabetic populations [6]. Contemporary guidelines from the National High Blood Pressure Education Program (NHBPEP) and the Eighth Joint National Committee (JNC) advocate the use of RAAS inhibitors (RAASi) in diabetic patients with hypertension to mitigate the risk of renal complications [10]. 

Recent advances in DN management have been catalyzed by the emergence of sodium-glucose cotransporter-2 inhibitors (SGLT2i). These agents exert renoprotective effects independent of their glucose-lowering action, including reductions in UAE and attenuation of glomerular hyperfiltration. Landmark clinical trials have demonstrated that SGLT2i preserve estimated glomerular filtration rate (eGFR) and delay progression to ESRD, even in individuals with impaired renal function at baseline [11,12,13]. As a result, the updated Kidney Disease: Improving Global outcomes (KDIGO) and the American Diabetes Association (ADA) guidelines now recommend the combined use of RAASi and SGLT2i in patients with DN [11,12]. The synergistic benefits of this combination reflect a shift toward multifactorial intervention strategies in DN, as emphasized by the American Heart Association’s recent scientific statement on integrated cardio-renal risk reduction in diabetes [14].

Although ACEi and ARBs are often used interchangeably, their side effect profiles remain a decisive factor in therapeutic selection. ACEi may induce dry coughs or angioedema, conditions largely absent in ARB treatment. Moreover, ACEi have been linked to a higher incidence of hyperkalemia, particularly in elderly or multi-morbid patients [15,16]. In contrast, ARBs are often preferred in clinical practice for their improved tolerability, which enhances long-term adherence and real-world effectiveness. 

Given the rising global burden of diabetes-associated ESRD and the evolving pharmacologic landscape, there is a critical need to evaluate optimized therapeutic combinations. In this study, we compare the effects of ARB monotherapy versus the combination of SLGT2i with ACEi (SGLT2/ACEi) on CKD progression in patients with diabetes. We specifically focus on their potential to mitigate the detrimental impact of chronic hyperglycemia and hypertension on renal outcomes.

## 2. Results

Data from 126 DM patients (*n* = 72 males, *n* = 54 females) were retrospectively reviewed over a two-year period at a private medical practice. The median age of participants was 62 years (range: 27–85 years). Baseline demographic and clinical characteristics are presented in Table 1. No modifications were made to patients’ existing treatment regimens, and no direct clinical interactions occurred during data extraction. Patients were categorized into two primary treatment groups: those receiving ARB monotherapy and those prescribed combination therapy with SGLT2i/ACEi.

### 2.1. Physiological and Biochemical Markers: One-Sample t-Test

**Figure 1:**
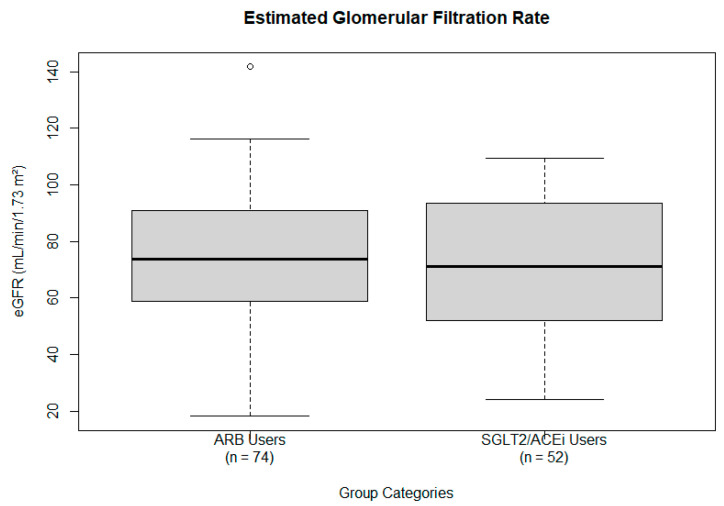
Estimated glomerular filtration rate (measured in mL/min/1.73 m^2^) by treatment group: ARB users (**left**) and SGLT2/ACE users (**right**). The box shows the IQR, whiskers indicate the range excluding outliers, and the solid dark line denotes the median estimated glomerular filtration rate. Several outliers are visible in both groups.

eGFR. A one-sample *t*-test was conducted using a hypothesized population mean of 107.5 mL/min/1.73 m^2^, derived from the average of the normal eGFR range (90 to 125 mL/min/1.73 m^2^) [17]. The observed sample mean eGFR was 73.37 ± 24.22 mL/min/1.73 m^2^. The test yielded a statistically significant result (t = −14.092, *p* = 2.137 × 10^−25^; 95% CI: 68.62 to 78.11), indicating that the sample’s average eGFR was significantly lower than the hypothesized mean. This suggests a deviation from normal kidney function in the population. However, this analysis does not imply preservation or deterioration of renal function over time, as no pre-treatment baseline values were available for longitudinal comparison. eGFR is also presented graphically in a boxplot (Figure 1), showing the interquartile range (IQR), whiskers excluding outliers, and the median.

**Figure 2:**
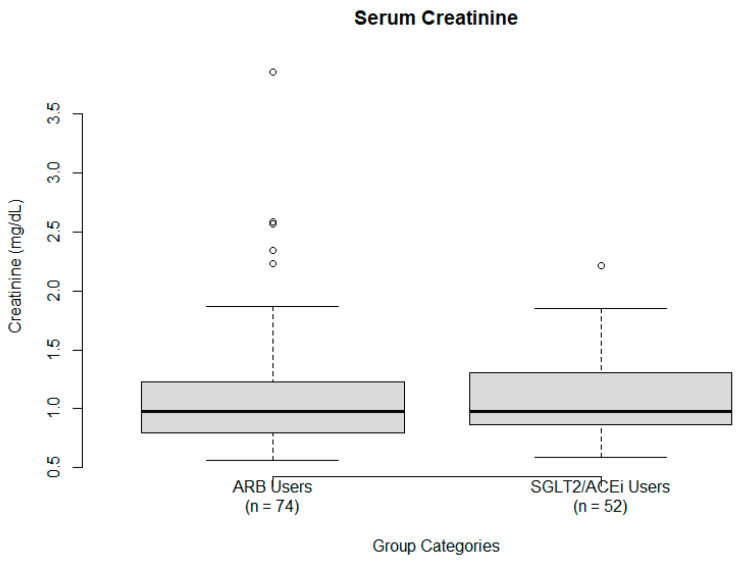
Serum creatinine levels (mg/dL) by treatment group: ARB users (**left**) and SGLT2/ACE users (**right**). The box shows the IQR, whiskers indicate the range excluding outliers, and the solid dark line denotes the median creatinine level. Several outliers are visible in both groups.

sCr. A one-sample *t*-test was performed using a hypothesized population mean of 0.87 mg/dL, based on reported normal values [18]. The observed sample mean sCr was 1.10 ± 0.48 mg/dL, yielding a statistically significant result (t = 4.7689, *p* = 6.381 × 10^−6^; 95% CI: 1.01 to 1.19). This indicates that the sample mean was significantly higher than the expected population mean, suggesting mildly reduced kidney function. Nevertheless, the values remain within clinically acceptable limits for most patients. sCr is visualized using a boxplot (Figure 2).

**Figure 3:**
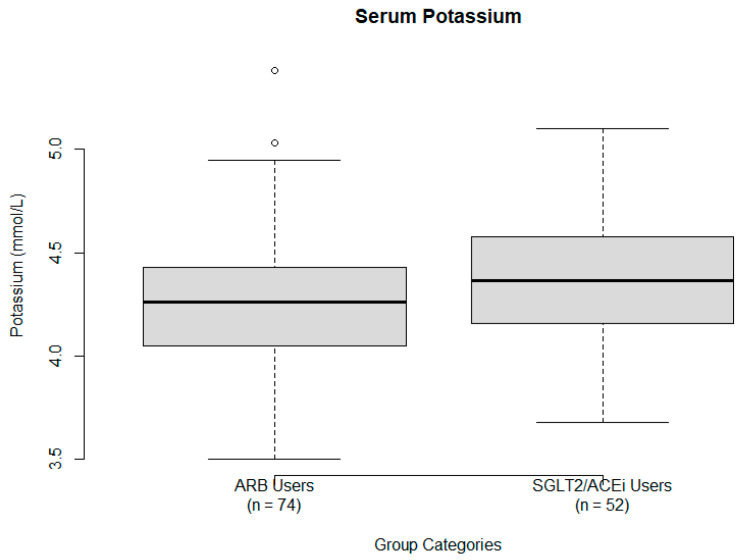
Serum potassium levels (mEq/L) by treatment group: ARB users (**left**) and SGLT2/ACEi users (**right**). Boxes represent the IQR, whiskers indicate the range, and the solid dark line represents the median potassium level. Outliers are shown as individual points.

Serum Potassium (K^+^). A one-sample *t*-test was conducted using a hypothesized population mean of 4.2 mEq/L, derived from the average of the reference range (4.0 to 4.4 mEq/L) [19]. The sample mean was 4.31 ± 0.33 mEq/L. A statistically significant result was obtained (t = 3.371, *p* = 0.00107; 95% CI: 4.25 to 4.38), indicating that ARB treatment may be associated with mildly elevated serum K^+^ levels, though values remained within the normal range. Boxplot analysis of K^+^ levels is provided in Figure 3.

**Figure 4:**
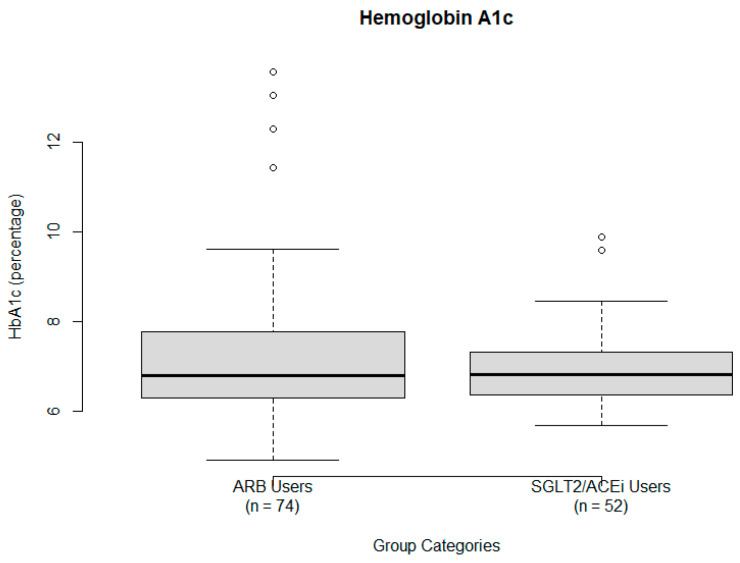
Hemoglobin A1c levels (percentage) by treatment group: ARB users (**left**) and SGLT2/ACEi users (**right**). The box represents the IQR, whiskers show the full data range excluding outliers, and the solid dark line represents the median HbA1c level. Several outliers are visible in both groups.

Hemoglobin A1c (HbA1c). A one-sample *t*-test was performed using a hypothesized population mean of 6.5%, based on the diagnostic threshold for DM [20]. The sample mean HbA1c was 7.23 ± 1.54. The test yielded a statistically significant result (t = 4.742, *p* = 7.105 × 10^−6^; 95% CI 6.93 to 7.53), suggesting that mean HbA1c levels in the sample exceeded the diabetic threshold. This indicates that ARB treatment alone may not sufficiently control HbA1c levels. HbA1c data are illustrated via a boxplot in Figure 4.

**Figure 5:**
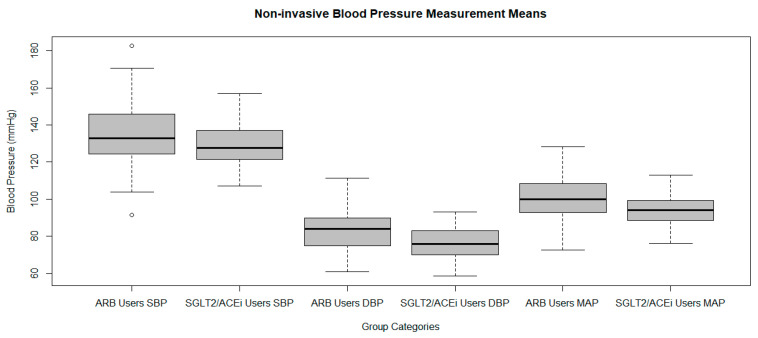
Non-invasive blood pressure (BP) measurement means by treatment group. From left to right: (1) active ARB treatment group: systolic blood pressure (SBP), (2) SGLT2/ACEi treatment group: SBP, (3) active ARB treatment group: diastolic blood pressure (DBP), (4) SGLT2/ACEi treatment group: DBP, (5) active ARB treatment group: mean arterial pressure (MAP), and (6) SGLT2/ACEi treatment group: MAP.

Systolic Blood Pressure (SBP). A one sample *t*-test was conducted using a hypothesized population mean of 120 mmHg, based on JNC-8 guidelines for normotension [10]. The sample mean SBP was 133.1 ± 16.2 mmHg. The test showed a statistically significant difference (t = 8.0926, *p* = 1.520 × 10^−12^; 95% CI: 129.9 to 136.3), indicating that SBP levels were significantly higher than the normotensive reference. Although elevated, these values suggest partial blood pressure (BP) control under ARB treatment. SBP distribution is shown in a boxplot (Figure 5).

### 2.2. Physiological and Biochemical Markers: Analysis of Variance (ANOVA)

To assess the relationship between eGFR and clinical biomarkers, an ANOVA was conducted. The predictors included HbA1c, K^+^, sCr, and albuminuria. Several ANOVA models were applied to the overall cohort (Table 2), the subgroup receiving ARB therapy (Table 3), and the subgroup receiving SGLT2i/ACEi-specific therapy (Table 4).

In the ARB group (*n* = 74), eGFR trends remained stable in 57 patients (77.0%), decreased in 7 patients (9.5%), and increased in 10 patients (13.5%) over the study period. Similarly, in the SGLT2i/ACEi group (*n* = 52), eGFR remained stable in 35 patients (67.3%), decreased in 6 patients (11.5%), and increased in 11 patients (21.2%). sCr values remained within normal limits and appropriately reflected changes in eGFR. As expected in CKD, we observed an inverse correlation between eGFR and sCr.

**Figure 6:**
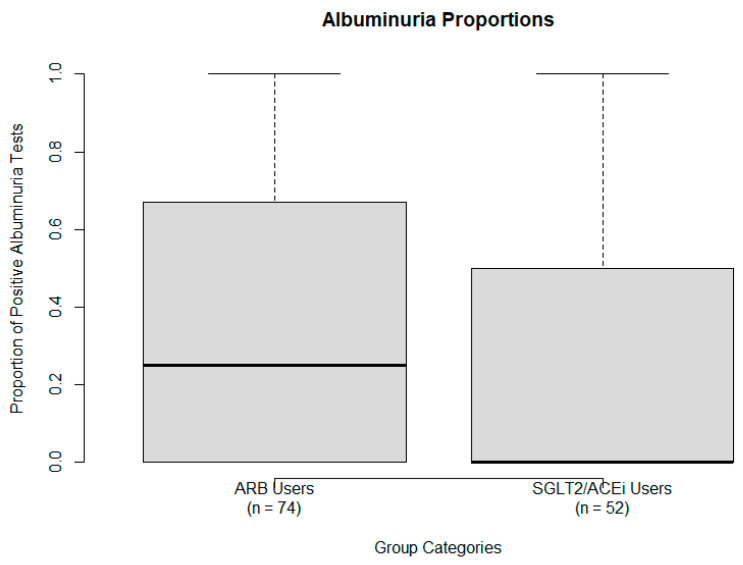
Proportion of positive albuminuria (≥30 mg/g) results by treatment group: ARB users (**left**) and SGLT2/ACEi users (**right**). Results are binary (0 = negative/trace, 1 = positive). Boxes represent the IQR, whiskers indicate full spread, and the solid line the median.

Among ARB users, 8 patients (10.8%) had persistently positive albuminuria, 32 patients (43.2%) had persistently negative results, 11 patients (14.9%) had positive averages, 13 patients (17.6%) had negative averages, and 10 patients (13.5%) had mixed outcomes. The proportion of positive albuminuria (≥30 mg/g) results by treatment group is illustrated in Figure 6. In the SGLT2i/ACEi group, 6 patients (11.5%) showed persistently positive results, 31 patients (59.6%) had persistently negative results, and the remainder had mixed or intermediate patterns.

Using the National Kidney Foundation’s (NKF) urine ACR risk classification [21], the distribution was as follows: (1) ARB group (*n* = 74): 39 (52.7%) low risk, 21 (28.4%) moderately increased risk, 8 (10.8%) high risk, 6 (8.1%) very high risk, 0 (0.0%) highest risk; and, (2) SGLT2i/ACEi group (*n* = 52): 28 (53.8%) low risk, 12 (23.1%) moderately increased risk, 9 (17.3%) high risk, 2 (3.8%) very high risk, 1 (1.9%) highest risk.

These findings suggest beneficial effects of ARB and SGLT2i/ACEi treatments in stabilizing eGFR and limiting MAU. Notably, albuminuria and eGFR deviations are independent predictors of CKD progression and mortality [9,22,23], with high albuminuria and low eGFR posing the highest risk of progression to ESRD.

## 3. Discussion

DN is driven by chronic hyperglycemia, which induces oxidative stress and inflammation, leading to structural and functional renal impairments. Hyperglycemia also activates the RAAS, contributing to glomerular disease, renal fibrosis, and CKD [24]. Angiotensin II, the active effector of the RAAS, promotes vasoconstriction, elevated BP, and extracellular matrix deposition, further exacerbating nephropathy. RAASi, including ARBs, have demonstrated efficacy in delaying CKD progression, reducing MAU, and stabilizing eGFR [24]. These benefits persist even in advanced CKD, although risk of hyperkalemia can limit their widespread use [24].

DN is frequently characterized by early glomerular hyperfiltration and MAU, both of which predict long-term renal decline. Since eGFR serves as a surrogate marker for kidney function, its trajectory over time is a critical endpoint in DN progression [19]. In our study, ARB therapy was associated with stabilization (and in some cases, slight improvement) of eGFR, suggesting attenuation of hyperfiltration-related damage. We assessed nephroprotection across three key biomarkers: (1) eGFR; (2) sCr; and (3) MAU. Importantly, longitudinal eGFR trends may yield more prognostic value than single-point measurements, especially in patients with heterogenous CKD trajectories [25]. While eGFR is a foundational tool for monitoring DN, refined formulas developed by the Chronic Kidney Disease Epidemiology Collaboration (CKD-EPI) aim to improve accuracy in DM populations [26]. Consistent with the NKF Kidney Disease Outcomes Quality Initiative (KDOQI) guidelines, both eGFR and albuminuria are essential for risk stratification and therapeutic decision-making [27].

The nephroprotective effects of ARBs are well documented and primarily linked to reductions in intraglomerular pressure and proteinuria. In our cohort, ARB treatment appeared to prevent further renal decline without significantly reversing kidney injury, suggestions disease stabilization rather than regression (i.e., causative reversal properties).

Hyperkalemia is a well-recognized concern with ARB use in DM patients. However, in our sample, serum K^+^ levels remained within normal limits, indicating general treatment tolerability. While not statistically significant (*p* = 0.071), the trend toward elevated K^+^ merits clinical attention given reports of hyperkalemia in 50% of patients receiving ARB therapy [16]. Recent studies highlight the need for individualized RAAS titration strategies in CKD to optimize efficacy while minimizing adverse events [24].

Glycemic control, as reflected by HbA1c, did not change significantly with ARB therapy, consistent with the known lack of glucose-lowering effect. This highlights the need for adjunctive strategies in achieving glycemic targets.

Notably, SBP was slightly lower in the SGLT2i/ACEi group compared to the ARB group. This could reflect sampling variability or differences in adherence to follow-up care and dietary recommendations. Additionally, use of concurrent antihypertensive agents, such as beta-blockers or calcium channel blockers, was not fully captured in the dataset and may have confounded results.

SGLT2i/ACEi combination therapy has emerged as an alternative and complementary approach in DN management. SGLT2i confer multiple metabolic and hemodynamic benefits, including BP reduction, weight loss, and cardiovascular risk mitigation [21]. However, these agents increase urinary glucose excretion and carry higher risks for urinary tract infections (UTIs), genital infections, and volume depletion. In patients at risk for osmotic complications or diabetic ketoacidosis (DKA), ARBs may be the safer option. Mechanistically, SGLT2i reduce intraglomerular pressure via tubuloglomerular feedback and promoting natriuresis, which could explain the greater sCr-lowering trends observed [28].

Landmark trials such as the Empagliflozin, Cardiovascular Outcomes, and Mortality in Type 2 Diabetes (EMPA-REG OUTCOME) [29] and the Dapagliflozin and Prevention of Adverse Outcomes in Chronic Kidney Disease (DAPA-CKD) [30] have demonstrated the renoprotective effects of SGLT2i in both diabetic and non-diabetic populations. In our analysis, SGLT2i/ACEi users showed slightly higher eGFR and lower MAU than the overall cohort, consistent with nephroprotection. Elevated HbA1c levels in this group may reflect indication bias, as SGLT2i are often prescribed to patients with poorer glycemic control.

While outcome differences between ARB monotherapy and SGLT2i/ACEi combination therapy were not statistically significant, the observed trends are clinically relevant. Recent CKD-EPI guidelines [27] recommend RAASi initiation with the addition of SGLT2i in proteinuric CKD patients with eGFR ≥ 25 mL/min/1.73 m^2^. Long-term data from DAPA-CKD support the additive benefit of dapagliflozin in patients on background RAAS blockade [30]. Vejakama et al. [31] noted the preferential use of ARBs over ACEi in patients at risk for cough or angioedema. Furthermore, the Ongoing Telmisartan Alone and in Combination with Ramipril Global Endpoint Trial (ONTARGET) demonstrated that ARBs offer equivalent cardiovascular and renal protection compared to ACEi, with a more favorable side-effect profile in high-risk patients [32]. Although ACEi and ARBs have comparable nephroprotective efficacy, ACEi are associated with persistent dry cough in up to 20% of users and increased risk of angioedema due to bradykinin accumulation. These factors may limit ACEi adherence and favor ARB use in certain populations.

Our cohort findings indicate that ARB therapy was associated with stabilization of both eGFR and sCr levels in the majority of patients, supporting its established role in long-term nephroprotection and aligning with existing literature. In cases where patients are intolerant to ARBs and ACEi remain the first-line RAASi, SGLT2i therapies may serve as a viable alternative. Although SGLT2i may cause initial decline in eGFR, both ARBs and ACEi have demonstrated greater consistency in maintaining renal function over time. This distinction is particularly relevant in DN management, where sustained preservation of kidney function remains a key therapeutic objective. Ultimately, the choice of pharmacologic intervention should be individualized based on patient-specific tolerabiliy, comorbidities, and the overall risk–benefit profile.

In our analysis, patients receiving ARB monotherapy demonstrated more stable eGFR levels compared to those treated with the SGLT2i/ACEi combination (Table 5; *p* = 3.148 × 10^−15^ vs. 2.002 × 10^−14^), supporting our hypothesis that ARBs may offer superior RAASi-mediated nephroprotection. However, a greater reduction in sCr was observed in the SGLT2i/ACEi group (Table 5; *p* = 3.241 × 10^−5^ vs. 1.481 × 10^−4^), which may reflect the enhanced osmotic and volume regulatory properties of SGLT2i compared to ARBs. These finding suggest a differential mechanism of renal benefit between treatment regimens. Nevertheless, longer follow-up and head-to-head monotherapy studies will be essential to better characterize and contextualize these trends. Future investigations employing larger, multivariable-adjusted models are warranted to elucidate these nuanced yet clinically relevant differences. It is also important to note that the renoprotective effect of SGLT2i appears to be potentiated when used in conjunction with ACEi, rather than as monotherapy.

### 3.1. Study Limitations

This study analyzed data from a cohort of 126 patients, with group assignment and sampling conducted using a convenience sampling approach. While this facilitated timely recruitment and data collection, it inherently introduced a risk of selection bias. As detailed in Section 4.1, the sample primarily included patients who demonstrated high adherence to their prescribed therapies and regularly attended clinical follow-up appointments. Consequently, the findings may not be generalizable to broader population with variable adherence patterns, inconsistent access to care, or different health-seeking behaviors. Patients who were non-compliant with treatment protocols or who missed follow-up appointments may exhibit different renal outcomes, and thus are underrepresented in this analysis.

Although both treatment and comparison groups were included, the relatively small sample size limits statistical power, potential obscuring subtle but clinically relevant difference between ARB monotherapy and SGLT2i/ACEi combination therapy. The variability observed in outcome measures such as eGFR, sCr, and MAU may also reflect differences in treatment regimens, including dosing heterogeneity. For example, while two patients may have been prescribed the same ARB (e.g., olmesartan), difference in dosage (20 mg once daily versus 20 mg twice daily) could have influenced pharmacodynamic outcomes, which were not stratified in this analysis. Furthermore, the study duration of approximately two years constrains evaluation of long-term therapeutic outcomes. DN is a progressive and chronic condition, and while short-term stabilization of renal function is encouraging, it remains unclear whether these effects are sustained or whether potential adverse effects, such as delayed-onset hyperkalemia, may emerge with prolonged ARB exposure. Longer observational studies are warranted to more definitively characterize the trajectory of renal function under chronic RAAS blockade.

As a retrospective analysis, this study lacked randomization and was subject to inherent confounding. Although relevant clinical variables were included, not all comorbidities that could impact renal function (e.g., cardiovascular disease [CVD], hypertension sensitivity, or concomitant nephrotoxic medication use) were controlled. Treatment durations varied across patients, and pre-treatment baseline values were not consistently available which limits the ability to draw causal inferences about treatment efficacy. The absence of uniform initiation points for therapy further complicates comparisons across groups and timepoints. 

These limitations highlight the need for prospective randomized studies with larger and more diverse population, standardized treatment protocols, and extended follow-up periods. Such efforts would provide more definitive insights into the comparative effectiveness in DM patients.

### 3.2. Future Implications and Perspectives

ARB therapy appears to be a safe and effective strategy for preserving renal function in DM patients, potentially mitigating disease progression and reducing the risk of CKD and eventual transition to ESRD. Nevertheless, careful clinical monitoring of eGFR, sCr, and serum K^+^ levels remain essential to ensure optimal management and mitigate adverse effects. Although ARB therapy may contribute to renal preservation, adjunctive interventions targeting BP and glycemic control are recommended to support comprehensive disease management. Longitudinal tracking of eGFR and sCr values may further aid clinicians in evaluating therapeutic effectiveness over time.

Our findings support the clinical relevance of ARB monotherapy and SGLT2i/ACEi combination therapy in the management of renal function in DM patients. Notably, the renal biomarker trends observed in this study suggest that ARB monotherapy may offer modest advantages in renal stability for select patient populations. These findings warrant further investigation through prospective, randomized controlled trials designed to evaluate long-term renal and cardiovascular outcomes across treatment modalities. Future studies may also benefit from examining pharmacodynamic, pharmacogenomic, and sociodemographic factors that could underlie differential treatment response.

Importantly, this study does not aim to establish the superiority or inferiority of any particular therapeutic regimen. Instead, it seeks to describe real-world clinical outcomes within a diverse patient population, where treatment decisions are often influenced by factors such as cost, comorbidities, and access to emerging therapeutic options. The treatment patterns observed reflect those encountered in primary care and community-based clinical settings, emphasizing the need to understand how existing therapies perform outside the controlled environment of randomized trials. 

As an exploratory, pilot investigation conducted at a single center, this study did not include patients receiving combined ARB/SGLT2i therapy due to their limited representation in the dataset. However, future research with a larger, more inclusive cohort is planned to incorporate this subgroup and facilitate more granular comparitive analyses between monotherapy and combination treatment approaches. Ultimately, advancing the management of DN and CKD in patients with DM will require individualized, patient-centered strategies that balance efficacy, safety, and contextual patient factors. Such efforts will be pivotal in slowing progression to CKD and reducing the burden of ESRD.

## 4. Methods

### 4.1. Study Design and Patient Selection

This retrospective, single-center, cohort study was conducted over a two-year period to evaluate the renal outcomes of different therapeutic regimens in patients with DM. Eligible participants were adults (≥18 years) with a confirmed diagnosis of T2DM, coexisting hypertension, and evidence of CKD stages 1–4. Inclusion criteria required the availability of at least two follow-up measurements of sCr and eGFR within the study period. Patients were excluded if they were ≤18 years, pregnant or breastfeeding, had a diagnosis of type 1 diabetes mellitus (T1DM), ESRD, or were receiving dialysis at baseline. Additional exclusion criteria included incomplete or missing medication records, prescription of other RAAS-modulating agents (e.g., direct renin inhibitors), active malignancy, advanced hepatic disease, or current enrollment in investigational drug trials. Patients receiving concomitant medications such as antihypertensives (e.g., beta-blockers, diuretics) and hypoglycemics (e.g., metformin, insulin) were included. These therapies were documented and considered during analysis to account for potential confounding effects. The presence of comorbidities such as hypertension, CVD, or isolated CKD did not constitute exclusion criteria and was permitted in both treatment groups. 

A convenience sampling approach was employed, identifying patients who actively attended follow-up appointments during the study period. To ensure sufficient longitudinal data, only patients with a minimum of three clinical follow-up visits were included. This method, while practical, may introduce selection bias, as patients with more consistent follow-up may differ systematically from those with less engagement in routine care. The treatment cohort consisted of patients with DN who had received ARB monotherapy for at least 12 months. The comparator group comprised patients that were prescribed a combination of SGLT2i/ACEi over a similar period. Data were extracted retrospectively from electronic medical records. No modification to clinical care or treatment protocols were made. It is important to note that some patients had normal ACR values at the time of data collection but had prior diagnoses of MAU and remained under nephrological surveillance. Their inclusion reflects real-world clinical practice, where biomarker levels may improve in response to treatment. This study protocol was approved by a formal data agreement form. Written informed consent was waived in accordance with institutional review policies, given the retrospective nature of the study, the use of de-identified data, and the absence of any direct patient interaction or intervention.

### 4.2. Clinical Parameters and Laboratory Measures

Clinical and laboratory data were retrospectively extracted from the electronic medical records of a private medical practice located in Bayonne, New Jersey, USA. Data included physiological and biochemical parameters routinely collected through bloodwork and urinalyses, namely eGFR, sCr, K^+^, HbA1c, MAU, and non-invasive BP measurements. These parameters were recorded at multiple time points throughout the two-year study period to assess longitudinal changes associated with the therapeutic regimens under investigation. Reference ranges for each laboratory parameter followed standard clinical guidelines, allowing for minor inter-laboratory variation. CKD stages were classified according to the NKF KDOQI eGFR staging criteria, while MAU was categorized using the NKF’s A1, A2, and A3 albuminuria classification system [22]. HbA1c was interpreted using ADA standards, with values <5.7% considered normal and ≥6.5% indicative of DM. As all included patients had pre-existing T2DM, HbA1c values were reported longitudinally but not reclassified diagnostically.

sCr levels were considered within normal range at 0.8–1.3 mg/dL, and normokalaemia was defined as serum K^+^ concentration between 3.5–5.0 mEq/L. BP was assessed according to JNC guidelines. According to the JNC-7 [33], a normal SBP was defined as 120–129 mmHg, and a diastolic blood pressure (DBP) as 80–84 mmHg, with corresponding mean arterial pressure (MAP) values ranging from of 93–99 mmHg. According to JNC-8 [10] recommendations, patients aged ≥60 years, or those with comorbid DM or CKD, were considered hypertensive if their BP measured ≥140/90 mmHg. MAU was initially categorized as a binary variable to facilitate consistency and reduce inter-laboratory variability: “0” indicated negative or trace albuminuria (<30 mg/g), and “1” indicated positive albuminuria (≥30 mg/g), based on urine ACR. To further capture clinical nuance and longitudinal variation in UAE trends, albuminuria was additionally stratified into five descriptive categories based on repeated measurements over the study period. These categories were (1) persistently positive (all UAE measurements were ≥30 mg/g), (2) persistently negative (all UAE measurements were <30 mg/g), (3) on-average positive (some negative values present, but the majority were ≥30 mg/g), (4) on-average negative (some positive values present, but the majority were <30 mg/g), and (5) on-average mixed results (equal or near-equal distribution of positive and negative results across timepoints; e.g., two of four measurements were ≥30 mg/g, and two were <30 mg/g). This extended classification scheme was developed in response to inconsistencies in UAE reporting across laboratories and provided a structured means to evaluate changes over time while maintaining clinical interpretability.

### 4.3. Statistical Analysis

Statistical analyses were performed to assess differences in physiological and biochemical markers between patients treated with ARB monotherapy and those receiving combination therapy with SGLT2i/ACEi. Independent sample *t*-tests were performed to compare continuous variables (e.g., eGFR, sCr, K^+^, HbA1c, SBP, DBP) between the two treatment groups. To control for potential confounding variables and to assess interactions between covariates, multivariate ANOVA were conducted. Variables examined in the ANOVA models included eGFR, HbA1c, and MAU status. MAU was analyzed both as binary outcome and across the five descriptive categories previously described.

Descriptive statistics, including mean, median, standard deviation (SD), and range, were computed for all continuous variables. All data were evaluated for parametric assumptions including normality and homogeneity of variance. When assumptions were met, values were expressed as mean ± SD. For categorical variables, frequency distributions and proportions were reported. To address the issue of multiple comparisons and mitigate the risk of Type I error, a Bonferroni correction was applied, setting the significance threshold at *p* < 0.01. In addition to *p*-values, effect sizes (Cohen’s *d*) were computed to quantify the magnitude of between-group differences, providing an estimate of practical significance. A priori power analysis was conducted to confirm that the sample size was sufficient to detect clinically meaningful difference with an alpha of 0.05 and power of 0.80. All statistical analyses were conducted using R software (version 2024.12.1; R Foundation for Statistical Computing, Vienna, Austria). Box plots and other graphical visualizations were generated to illustrate between-group trends and distributions across clinical parameters.

## 5. Conclusions

Both ARB monotherapy and SGLT2i/ACEi combination therapy demonstrate promise in preserving renal function, slowing the progression of DN, and reducing the risk of CKD and eventual ESRD. These treatment strategies offer complementary nephroprotective benefits; however, their effectiveness may vary depending on patient-specific factors such as comorbid conditions, medication tolerability, and treatment adherence. The optimal management of DN requires a personalized, multifactorial approach that integrates pharmacologic interventions with routine monitoring of renal, glycemic, and cardiovascular parameters. Our findings support the clinical utility of ARB monotherapy, particularly for patients who may not tolerate or have limited access to newer agents. These results contribute to the expanding evidence base for ARBs in real-world clinical settings. However, the study’s retrospective design, short observation period, and modest sample size limit the generalizability of its findings. Future research should prioritize prospective, multicenter investigations to assess the long-term renal and cardiovascular outcomes with ARB therapy, including its role in combination with emerging agents such as SGLT2i. Such studies are essential to define optimal treatment algorithms and improve outcomes in this high-risk population.

## Figures and Tables

**Table 1:** Baseline characteristics of diabetic patients with categorization based on active angiotensin receptor blocker (ARB) prescriptions/treatment (Rx) or SGLT2 and ACE inhibitor (SGLT2i/ACEi) prescriptions/treatment.

Characteristic(s)	Totals	Active ARB Rx	SGLT2i/ACEi Rx
*n* = 126		74	52
Age			
Mean (years)	61.8 ± 11.0	62.1 ± 10.16	61.5 ± 12.11
Sex			
Female	54	29	25
Male	72	45	27
Insulin status			
Insulin-dependent	20	12	8
Non-insulin dependent	106	62	44
Race/Ethnicity			
Black, non-Hispanic	47	33	14
Hispanic	37	19	18
Asian	29	16	13
White	13	6	7
Chronic kidney disease (CKD) stage			
No CKD/CKD1	52	32	20
CKD2	38	23	15
CKD3a/3b	32	16	16
CKD4	4	3	1
CKD5	0	0	0

**Table 2:** ANOVA results for overall sample (*n* = 101).

Predictor	df	SS/MS *	F-Value	*p*-Value
HbA1c	1	1224	5.276	0.0236
Potassium	1	2783	12.037	0.0008
Creatinine	1	31,800	137.540	<2.0 × 10^−16^
Albuminuria	1	301	1.301	0.2569
Residuals	95	21,964		

* Sum of squares (SS) and mean squares (MS) were equal for each predictor.

**Table 3:** ANOVA results for ARB-treated subgroup (*n* = 74).

Predictor	df	SS/MS *	F-Value	*p*-Value
HbA1c	1	610	2.706	0.1045
Potassium	1	1956	8.676	0.0044
Creatinine	1	23,813	105.629	1.49 × 10^−15^
Albuminuria	1	108	0.479	0.4912
Residuals	69	15,555		

* SS and MS were equal for each predictor.

**Table 4:** ANOVA results for SGLT2i/ACEi-treated subgroup (*n* = 52).

Predictor	df	SS/MS *	F-Value	*p*-Value
HbA1c	1	1915	13.895	0.0005
Potassium	1	558	4.045	0.0500
Creatinine	1	20,563	149.186	3.44 × 10^−16^
Albuminuria	1	941	6.830	0.0120
Residuals	47	6478		

* SS and MS were equal for each predictor.

**Table 5:** One-sample *t*-test results comparing mean values, standard deviations (SD), *t*-statistics, *p*-values, and Cohen’s *d* effect sizes across physiologic and biochemical markers.

Marker		Mean ± SD	*t*-Statistic	*p*-Value	Effect Size (*d*)
eGFR (mL/min/1.73 m^2^)	Overall	73.37 ± 24.22	−14.09	2.14 × 10^−25^	−1.41
	ARB Users	73.57 ± 24.00	−12.16	3.15 × 10^−19^	−1.41
	SGLT2/ACEi Users	71.74 ± 24.44	−10.55	2.00 × 10^−14^	−1.46
HbA1c (%)	Overall	7.23 ± 1.54	4.74	7.11 × 10^−6^	0.47
	ARB Users	7.36 ± 1.68	4.40	3.58 × 10^−5^	0.51
	SGLT2/ACEi Users	7.02 ± 0.92	4.08	1.60 × 10^−4^	0.57
Potassium (mEq/L)	Overall	4.31 ± 0.33	3.37	1.07 × 10^−3^	0.33
	ARB Users	4.27 ± 0.33	1.83	7.11 × 10^−2^	0.21
	SGLT2/ACEi Users	4.38 ± 0.30	4.36	6.30 × 10^−5^	0.60
Creatinine (mg/dL)	Overall	1.10 ± 0.48	4.77	6.38 × 10^−6^	0.48
	ARB Users	1.12 ± 0.53	4.00	1.48 × 10^−4^	0.47
	SGLT2/ACEi Users	1.10 ± 0.36	4.56	3.24 × 10^−5^	0.64
Systolic Blood Pressure (mmHg)	Overall	133.1 ± 16.2	8.09	1.52 × 10^−12^	0.81
	ARB Users	135.1 ± 17.2	7.59	8.15 × 10^−11^	0.88
	SGLT2/ACEi Users	129.1 ± 12.8	5.16	4.02 × 10^−6^	0.71

## Data Availability

This study was a retrospective analysis conducted at a single center using de-identified patient data. In accordance with institutional policy and to ensure compliance with the United States Health Insurance Portability and Accountability Act (HIPAA) of 1996, the underlying dataset is not publicly available.

## Abbreviations

The following abbreviations are used in this manuscript:

ACEi	angiotensin-converting enzyme inhibitors
ACR	albumin-to-creatinine ratio
ADA	American Diabetes Association
ANOVA	Analysis of variance
ARBs	angiotensin receptor blockers
BP	blood pressure
CKD	chronic kidney disease
CKD-EPI	Chronic Kidney Disease Epidemiology Collaboration
CVD	cardiovascular disease
DAPA-CKD	Dapagliflozin and Prevention of Adverse Outcomes in Chronic Kidney Disease
DBP	diastolic blood pressure
DKA	diabetic ketoacidosis
DM	diabetes mellitus
DN	diabetic nephropathy
eGFR	estimated glomerular filtration rate
EMPA-REG OUTCOME	Empagliflozin, Cardiovascular Outcomes, and Mortality in Type 2 Diabetes
ESRD	end-stage renal disease
HbA1c	hemoglobin A1c
IQR	interquartile range
JNC	Joint National Committee
K^+^	potassium
KDIGO	Kidney Disease: Improving Global Outcomes
KDOQI	National Kidney Foundation Kidney Disease Outcomes Quality Initiative
MAP	mean arterial pressure
MAU	microalbuminuria
MS	mean squares
NKF	National Kidney Foundation
NHBPEP	National High Blood Pressure Education Program
ONTARGET	Ongoing Telmisartan Alone and in Combination with Ramipril Global Endpoint Trial
RAAS	renin–angiotensin–aldosterone system
RAASi	renin–angiotensin–aldosterone system inhibitors
Rx	prescription/treatment
SBP	systolic blood pressure
sCr	serum creatinine
SD	standard deviation
SGLT2i	sodium-glucose cotransporter-2 inhibitors
SGLT2i/ACEi	sodium-glucose cotransporter-2 inhibitor/angiotensin-converting enzyme inhibitor
SS	sum of squares
T1DM	type 1 diabetes mellitus
T2DM	type 2 diabetes mellitus
UAE	urinary albumin excretion
UTIs	urinary tract infections
